# Plasma bilirubin levels are reduced in first-episode psychosis patients and associates to working memory and duration of untreated psychosis

**DOI:** 10.1038/s41598-021-87096-z

**Published:** 2021-04-06

**Authors:** Meneca Becklén, Funda Orhan, Fredrik Piehl, Simon Cervenka, Carl M. Sellgren, Lena Flyckt, Sophie Erhardt, Helena Fatouros-Bergman

**Affiliations:** 1 Department of Physiology and Pharmacology, Karolinska Institutet, 171 77 Stockholm, Sweden; 2 Neuroimmunology Unit, Department of Clinical Neuroscience, Karolinska Institutet, Stockholm, Sweden; 3 Centre for Psychiatry Research, Department of Clinical Neuroscience, Karolinska Institutet, & Stockholm Health Care Services, Region Stockholm, Stockholm, Sweden

**Keywords:** Diagnostic markers, Neuroscience, Biomarkers

## Abstract

Schizophrenia is a severe mental disorder and one of its characteristics is cognitive impairments. Findings regarding levels of the heme metabolite and plasma antioxidant bilirubin in schizophrenia are inconclusive. However, a recently published study indicate that low levels of bilirubin may be implicated in the memory impairments seen in the disorder. The aim of this cross-sectional study was to investigate the levels of bilirubin in individuals with a first-episode psychosis (FEP) and to examine if bilirubin levels were associated to cognitive impairments, symptoms and duration of untreated psychosis (DUP). Bilirubin levels were reduced in 39 individuals with FEP compared with 20 HC (median [IQR]: 11.0 [9.0–13.0] µM vs. 15.0 [11.5–18.5] µM). In individuals with FEP, bilirubin levels were also positively correlated to two working memory tests (r = 0.40 and r = 0.32) and inversely correlated to DUP (r = − 0.36). Findings were not influenced by confounding factors. The results confirm the antioxidant deficit previously seen in schizophrenia, but also indicate that these changes may be related to DUP. The study also confirms that bilirubin may be implicated in the cognitive deficits that accompanies the disorder, here for the first time presented in individuals with FEP.

## Introduction

Schizophrenia is a severe mental disorder with a lifetime prevalence of around 0.7%^1^. The patients exhibit positive, negative and general symptoms^2^. Cognitive impairments is a core feature of the disorder and have been demonstrated both in antipsychotic medicated^3^ and in drug-naïve patients^4^. Individuals with a first-episode psychosis (FEP) are experiencing the debut of acute psychosis that may be one of the early manifestations of schizophrenia. However, since the diagnosis of schizophrenia requires certain criteria regarding the intensity and duration of the psychosis, the diagnosis cannot be established in the early phase of the psychosis^5^.

Bilirubin is a metabolite resulting from the haemoglobin (Hb) catabolism to heme, the oxygen-carrying part of red blood cells (RBC), and is metabolised in the liver by the enzyme uridine diphospho-glucuronosyltransferase (UGT)^6^. UGT catalyses the conjugation of the water-insoluble fraction of the heme metabolite, i.e. unconjugated bilirubin (UCB), which is subsequently excreted into the bile. Transient bilirubin encephalopathy is the result of accumulation of bilirubin in the central nervous system (CNS) and is highly detrimental in the perinatal state^7^. On the other hand, bilirubin is an efficient endogenous plasma antioxidant with the ability to counteract oxidative stress^8^. Oxidative stress has been suggested to be part of the pathophysiology of schizophrenia^9–18^. Previous studies have indicated a relationship between peripheral and CNS oxidative stress parameters^19^ and blood components have been suggested to reflect the role of central oxidative stress in schizophrenia^20, 21^.

Studies have previously indicated that aberrations in bilirubin levels may affect cognitive capacity. Serum bilirubin levels have been positively correlated with cognitive scores in patients with mild cognitive impairment^22^ and subcortical ischemic vascular disease^23^. A preliminary finding recently published also found a positive correlation between low levels of bilirubin with immediate memory score in patients with schizophrenia^24^. Several studies have also demonstrated lower levels of bilirubin in these patients compared with HC^9, 12, 20, 24–26^. Reductions in endogenous antioxidants, such as bilirubin, seen in schizophrenia have been suggested to represent their action as sacrificial molecules while neutralizing reactive oxygen species (ROS)^27^. Free radicals have been suggested to be implicated in schizophrenia; possibly mediated via membrane pathology^21, 28^. The brain, which is rich in polyunsaturated fatty acids (PUFAs), is at particular risk of oxidative damage due to high oxygen consumption, high content of lipids, transition metals and neurotransmitters associated with schizophrenia^29^. PUFAs have previously been shown to be reduced in both central and peripheral membranes of patients with schizophrenia^29, 30^.

On the contrary, some studies have demonstrated elevated levels of bilirubin in individuals with schizophrenia compared with the general population or healthy controls (HC)^16, 31–33^. UCB may induce cell injury in microglia and astrocytes triggering an inflammatory response and the generation of ROS^34^. Elevation of bilirubin levels have been suggested to represent a functional upregulation of the antioxidant defence^35–37^ or RBC membrane fragility^27, 33, 38^ in conditions of oxidative stress in schizophrenia.

The aim of the present study was to investigate possible differences in levels of total plasma bilirubin between individuals with a first-episode psychosis (FEP) and matched group of HCs, as well as correlating bilirubin levels with cognition as well as symptom severity and duration of untreated psychosis.

## Material and methods

### Study protocol

Individuals with FEP and HC were recruited to the study in accordance with the latest Helsinki Protocol and approved by the Regional Ethics Committee in Stockholm (2010/879-31-1). The project is a part of Karolinska Schizophrenia Project (KaSP), a research consortium of multidisciplinary nature with the common goal of investigating the pathophysiology of schizophrenia. Details of the study design have previously been published elsewhere^39^. Participation was voluntary, did not affect treatment and required an oral and written consent. None of the participants was deemed as vulnerable and signed written informed consent forms were therefore obtained from all 64 participants. All participants underwent a physical examination, routine blood and urine testing, evaluation of medical history, a magnetic resonance imaging (MRI) scan and a lumbar puncture. Blood sampling was performed between 07.45–10.00 h in the majority of participants. Due to clinical routines, some individuals with FEP however underwent blood sampling between 10.30 and 13.15 h and to compensate for this, the same proportion of HC underwent blood sampling in the later time interval. All participants were instructed to avoid physical activity for the proceeding eight hours. Exclusion criteria were severe somatic or neurological illness, autism spectrum disorder and substance abuse other than tobacco use (smoking or snuff). This is evaluated by the use of Alcohol Use Disorder Identification Test and Drug Use Disorders Identification Test. In order to exclude the possible bias of several confounding factors affecting bilirubin levels, we investigate differences in demographic characteristics, antipsychotic medication, tobacco use, levels of the liver markers plasma (p) alanine transaminase (ALT), p aspartate transaminase (AST), p gamma-glutamyl transferase (GGT) as well as levels of p-albumin, blood (b) Hb and serum (s) cobalamin between individuals with FEP and HC.

### FEP

Forty-two individuals with FEP (27 males and 15 females) were recruited between March 2011 to January 2014 from psychiatric in- and outpatient and emergency wards in Stockholm, Sweden. Two of these wards were highly specialised units for individuals with FEP. The diagnostic and Statistical manual of Mental Disorder IV (DSM-IV) were used for initial diagnosis (n_1_) and in a 1.5-year re-evaluation (n_2_) of the patients. Included individuals with FEP met the criteria for schizophrenia (n_1_ = 12, n_2_ = 27), schizophreniform disorder (n_1_ = 15, n_2_ = 0), psychotic disorder not otherwise specified (n_1_ = 9, n_2_ = 4), delusional disorder (n_1_ = 3, n_2_ = 4), schizoaffective syndrome (n_1_ = 1, n_2_ = 3), brief psychotic disorder (n_1_ = 1, n_2_ = 1), severe depression with psychotic features (n_1_ = 1, n_2_ = 0) and no diagnosis (n_1_ = 0, n_2_ = 3).

43% of the patients had received short-time (less than a month in all cases but one who had a treatment period of almost two months) treatment with an antipsychotic agent at time of sampling (Table 1). Use of anxiolytics and sedatives were permitted and 31% of patients were naïve to all medications (Table 1). DUP was established by information from the patients or their relatives and were defined as the period between the debut of psychotic symptoms until initiation of treatment targeting the psychotic symptoms. Global Assessment of Functioning (GAF), the Positive and Negative Syndrome Scale (PANSS)^2^ and the Clinical Global Impression (CGI) were used for rating the patients regarding symptom severity, functioning dimensions and clinical characterisation. Measurements of bilirubin were available for analysis in 39 of the forty-two individuals with FEP. For details of this sub cohort see Supplementary Table S1 online.

**Table 1:** Demographic and clinical characteristics of the study population.

Characteristic	Median [Interquartile range] (n)		
	Healthy controls	FEP patients	*p* value
	(n = 22)	(n = 42)	
Gender (M:F)	11:11 (22)	27:15 (42)	0.269^a^
Age (years)	25.0 [22.0–28.0] (22)	27.5 [23.0–33.0] (42)	0.124^b^
BMI (kg m^−2^)	21.6 [20.7–23.8] (21)	22.2 [20.5–25.3] (40)	0.455^b^
Weight (kg)	67.9 [61.0–81.8] (18)	70.2 [62.5–78.3] (40)	0.658^b^
Length (cm)	173 [166–185] (19)	177 [170–181] (40)	0.759^b^
Tobacco (Y:N)	0:22; 0% (22)	12:27; 31% (39)	0.004^a^*
DUP (months)	–	4.5 [2.0–18.0] (38)	–
**Medication (Y:N)**			–
Antipsychotics	–	18:24; 43% (42)	–
Benzodiazepines	–	12:30; 29% (42)	–
Zopiclone	–	10:32; 24% (42)	–
Antidepressants	–	5:37; 12% (42)	–
Phenothiazine derivatives	–	11:31; 26% (42)	–
Antiepileptic’s	–	1:41; 2,4% (42)	–
Combination of > 2 drugs	–	16:26; 38% (42)	–
Totally drug-naive	–	13:29; 31% (42)	–
**PANSS**			
Positive	–	19 [15–24] (42)	–
Negative	–	13 [10–19] (42)	–
General	–	38 [31–45] (42)	–
Total	–	75 [60–83] (42)	–
Anxiety	–	4 [3–5] (42)	–
Depression	–	3 [2–4] (42)	–
**Level of functioning**			
GAF S	*–*	31 [28–36] (42)	*–*
GAF F	–	40 [35–50] (42)	–
GAF S HVLY	*–*	65 [40–75] (39)	*–*
GAF F HVLY	–	65 [55–79] (39)	–
CGI Score	*–*	4.0 [3.0–5.0] (42)	*–*

Abbreviations***:*** FEP, first-episode psychosis; M:F, Male:Female; Y:N, Yes:No; (n), number; BMI, body mass index; DUP, Duration of untreated psychosis; PANSS, Positive and Negative Syndrome Scale; GAF S, Global Assessment of Functioning Symptoms; GAF F, Global Assessment of Functioning Functions; HVLY, highest value last year; CGI, Clinical Global Impression. ^a^Pearson’s chi-squared test ^b^Mann-Whitney U-test, two-sided. *p value < 0.05.

### HC

Twenty-two HC (11 males and 11 females) were recruited by advertisement and matched with the individuals with FEP regarding gender and age. They underwent The Mini International Neuropsychiatric Interview (M.I.N.I.) for screening of psychiatric disorders. Additional exclusion criteria for HC were previous and current psychiatric illness or having a first-degree relative with a psychotic disorder. One of the HC had oligoclonal bands in the cerebrospinal fluid and signs of demyelinating disease on MRI. Due to the fact that the neurological examination was normal, this HC had test results in parity with other HC and had no ongoing or previous relevant neurological symptoms, the HC was not excluded from the cohort. Values of bilirubin were available in 21 of the twenty-two HC and after exclusion of an outlier twenty HC were included in the final analysis. For details of this sub cohort see Supplementary Table S1 online.

### Plasma isolation and bilirubin detection

Quantifications of albumin and bilirubin in plasma were performed using immunoturbidimetry and colorimetry respectively (Cobas 8000; Roche Diagnostics, Risch-Rotkreuz, Switzerland). All analyses were part of routine blood testing for the individuals with FEP and analysed on fresh samples. The peripheral blood extraction was executed with standard venepuncture techniques and all analyses were performed using routine operating procedures at Karolinska University Hospital (Stockholm, Sweden).

### Cognitive tests

All 64 participants were evaluated with The Measurement and Treatment Research to Improve Cognition in Schizophrenia (MATRICS) Consensus Cognitive Battery^40^. Previously published data on nearly the same cohort presented a reduced cognitive capacity in individuals with FEP compared with HC^39^. We here focused on the domains verbal learning (Hopkins Verbal Learning Test-Revised [HVLT-R]), working memory (Wechsler Memory Scale-3rd Edition [WMSIII]: Spatial Span [SS], Letter-Number Span [LNS]) and speed of processing (Trail Making Test: Part A [TMT-A], Brief Assessment of Cognition in Schizophrenia: Symbol Coding [BACS-SC], Category Fluency: Animal Naming [Fluency-AN]) of the MATRICS battery since a meta-analysis of cognitive deficits in drug-naïve individuals with FEP has shown that these domains had the largest impairments^4^.

### Statistical analysis

SAS software (version 9.4, SAS Institute Inc., Cary, USA) for Windows was used for statistical analysis. GraphPad Prism version 8.0 (GraphPad Software, Inc., San Diego, CA) was used for data presentation in forms of scattered plot graphs. Due to the relatively small sample size of the cohort, Shapiro–Wilk’s test were used in order to screen for normal distribution. Subsequently, the most accurate test of choice in each analysis was identified, based on parametric or non-parametric sample distribution, as indicated in the figure and tables. Pearson’s chi-squared test were used in the analysis of binary variables. Mann–Whitney U-test were used in the non-parametric comparisons of the cohorts. Student’s unpaired t-test (when equal variances) and Satterthwaite t-test (when unequal variances) were used in the parametric comparisons of the cohorts. Spearman’s rank correlations (non-parametric) were used in all correlation analysis. All *p* values are presented as two-sided and considered significant when *p* < 0.05.

Outliers were identified by schematic box plot analysis in SAS and defined as values below Q1 – 1.5 × IQR or above Q3 + 1.5 × IQR, where Q stands for quartile. One healthy control with a high bilirubin value of 52 µmol/l was identified as an outlier and omitted from analyses. Inclusion of this individual would have enhanced the differences in bilirubin levels between individuals with FEP and HC. This individual is however included in Table 1. For details of the sub cohort after exclusion of this outlier see Supplementary Table S1 online.

Only the cognitive tests that, after the Bonferroni correction (α-threshold of 0.00833 [0.05/6], differed significantly between individuals with FEP and HC (see Supplementary Table S2 online) were used for further correlation analysis with bilirubin levels.

## Results

### Demographics

Demographic and clinical characteristics (Table 1) has in part previously been published elsewhere^39^. In the full cohorts, a larger proportion of individuals with FEP were males compared with HC and the median age, weight, length and BMI were higher in the FEP cohort (Table 1). Thirty-one percent of the individuals with FEP used tobacco compared with none of the HC and this difference was statistically significant (*p* = 0.004). The same patterns were identified in the sub cohort only presenting patients with bilirubin values and excluding the outlier (n = 39, n = 20) (see Supplementary Table S1 online).

### Plasma bilirubin levels in individuals with FEP versus HC

**Figure 1:**
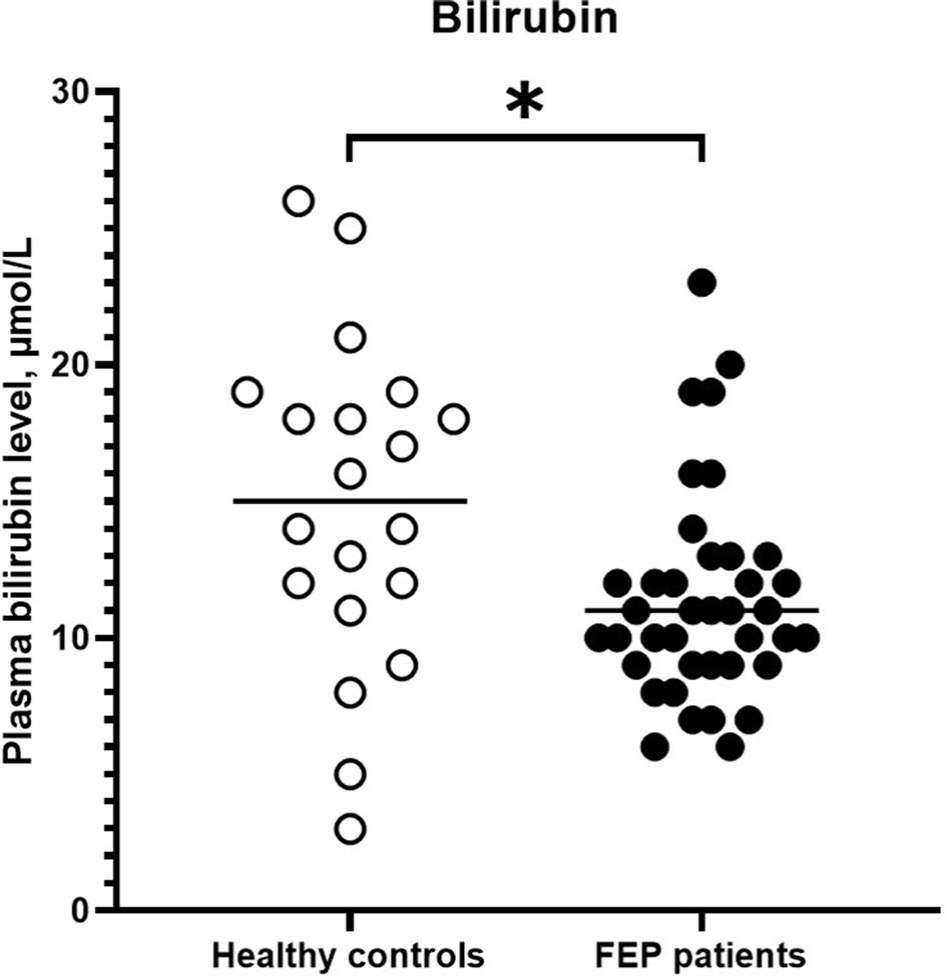
Levels of plasma bilirubin in HC versus individuals with FEP (HC: median 15.0 µM, IQR 11.5–18.5 µM, n = 20 vs. FEP: median 11.0 µM, IQR 9.0–13.0 µM, n = 39, *p* value 0.014). Horizontal lines represent the median for each group. Abbreviations: FEP, First episode psychosis. Statistical differences were determined using the Mann–Whitney U-test, two-sided. *p value < 0.05.

The bilirubin concentrations displayed in Fig. 1 were significantly lower in individuals with FEP compared with HC (median [IQR]: 11.0 [9.0–13.0] µM versus 15.0 [11.5–18.5] µM, *p* = 0.014). In individuals with FEP, we observed no significant differences in the levels of bilirubin in antipsychotic-treated and antipsychotic drug-naïve individuals with FEP (Table 2). Additionally, no differences between individuals with FEP on and off other psychotropic drugs or tobacco were found (Table 2). No associations were found between bilirubin levels and demographic factors in individuals with FEP (see Supplementary Table S3 online) or in HC except for one correlation between age and bilirubin in HC (see Supplementary Table S4 online).

**Table 2:** Plasma bilirubin with regard to medication and tobacco use in individuals with FEP.

Medication/tobacco	Median [Interquartile range] (n)		
	Plasma bilirubin levels (µmol/l)		*p* value^a^
	FEP patients on drug or on tobacco	FEP patients off drug or off tobacco	
Antipsychotics^b^	10.0 [10.0–13.0] (16)	11.0 [8.0–12.0] (23)	0.70
Antidepressants^c^	12.5 [10.0–16.5] (4)	10.0 [9.0–12.0] (35)	0.31
Antiepileptics^d^	8.0 (1)	11.0 [9.0–13.0] (38)	0.25
Benzodiazepines^e^	12.0 [9.0–16.0] (11)	10.5 [9.0–12.0] (28)	0.46
Zopiclone	11.0 [10.0–13.0] (10)	10.0 [9.0–12.0] (29)	0.18
Phenothiazine derivates^f^	10.0 [10.0–13.0] (11)	11.0 [9.0–12.5] (28)	0.73
Any treatments^g^	11.0 [9.0–13.0] (27)	10.5 [7.0–14.0] (12)	0.62
Tobacco	10.0 [9.5–11.5] (12)	11.0 [8.5–12.5] (24)	0.74

Abbreviations: FEP, First-episode psychosis; (n), number. ^a^Mann-Whitney U-test, two-sided. ^b^All antipsychotics combined. ^c^All antidepressants combined. ^d^All antiepileptic’s combined. ^e^All benzodiazepines combined. ^f^All Phenothiazine derivatives combined. ^g^Either N, AD, AE, BZD, Z, PD or a combination of these drugs.

### Cognitive performance in individuals with FEP versus HC and correlations between cognitive performance and bilirubin levels in individuals with FEP

Correlations between the cognitive tests, which were significantly reduced in individuals with FEP after adjustment for multiple testing [see Supplementary Table S2 online], and bilirubin levels are displayed in Table 3. Two positive correlations were identified between bilirubin levels and the working memory test SS (r = 0.40, *p* = 0.014) and LNS (r = 0.32, *p* = 0.048) of WMSIII. The correlation between bilirubin and WMSIII-SS remained significant (*p* < 0.05) in sub group analysis of the antipsychotic naïve individuals with FEP (see Supplementary Table S5 online). No correlations were found between bilirubin and working memory in HC (see Supplementary Table S6 online).

**Table 3:** Spearman’s rank correlation of plasma bilirubin levels with cognitive performance, clinical symptoms and DUP in individuals with FEP.

Characteristic	r-value	(n)	*p* value
**Cognitive tests**			
Verbal learning (HVLT-R)	− 0.0647	38	0.700
Working Memory (WMSIII-SS)	0.3964	38	0.014*
Working Memory (WMSIII-LNS)	0.3231	38	0.048*
Speed of processing (BACS-SC)	0.0555	38	0.741
**PANSS**			
Positive	0.0595	39	0.719
Negative	− 0.0041	39	0.980
General	− 0.0554	39	0.738
Total	− 0.0347	39	0.834
Anxiety	− 0.1216	39	0.461
Depression	− 0.1409	39	0.392
**Level of functioning**			
GAF S	− 0.1298	39	0.431
GAF F	− 0.1358	39	0.410
GAF S HVLY	− 0.0462	36	0.789
GAF F HVLY	− 0.0250	36	0.885
CGI Score	− 0.0800	39	0.628
DUP	− 0.3558	35	0.036*

Abbreviations: FEP, first-episode psychosis; (n), number; HVLT-R; Hopkins Verbal Learning Test—Revised; WMSIII-SS, Wechsler Memory Scale-3rd Edition—Spatial Span; WMSIII-LNS, Wechsler Memory Scale-3rd Edition – Letter-Number Span; BACS-SC, Brief Assessment of Cognition in Schizophrenia—Symbol Coding; PANSS, Positive and Negative Syndrome Scale; GAF S, Global Assessment of Functioning Symptoms; GAF F, Global Assessment of Functioning Functions; HVLY, highest value last year; CGI, Clinical Global Impression; DUP, Duration of untreated psychosis. *p value < 0.05.

### Correlations between bilirubin levels and symptoms and function in individuals with FEP

We observed no significant association between bilirubin and scores on PANSS, GAF and CGI (Table 3).

### Correlation of bilirubin levels and DUP in individuals with FEP

A significant inverse correlation (r = − 0.36, *p* = 0.036) between DUP and bilirubin levels was found and is displayed in Table 3.

### Levels of liver markers, p-albumin, b-Hb and s-cobalamin in individuals with FEP versus HC

We found no significant differences in levels of p-ALT, p-AST, p-GGT, p-albumin, b-Hb and s-cobalamin between individuals with FEP and HC (see Supplementary Table S7 online).

## Discussion

We here demonstrate lower levels of plasma bilirubin in individuals with FEP compared with HC. Notably, bilirubin levels were found to be positive correlated with working memory, a cognitive domain impaired in FEP^4^. Moreover, an inversed correlation between bilirubin and DUP was observed, such that lower bilirubin levels was associated with a longer DUP. The result of the present study is in agreement with studies finding decreased levels of bilirubin in schizophrenia.

These results provide support for the notion that schizophrenia is associated with oxidative stress due to increased free radical production^14, 21, 27^ and/or perturbations in the antioxidant defence system (AODS)^9, 11, 12, 16, 20, 25^. Lower levels of bilirubin in people with the diagnosis of schizophrenia has been suggested to be the result of a relative lack of antioxidants^37^ or an oxidative stress-induced bilirubin consumption^26^.

Equivalent to our findings, Yin et al. found reduced levels of bilirubin in two weeks antipsychotic-free patients with chronic schizophrenia compared with HC^24^. In line with our study, bilirubin was positively correlated with memory, although immediate memory, in individuals with schizophrenia but not in HC. The authors hypothesized that the memory impairment may be mediated by the antioxidant system defects resulting from decreased bilirubin concentrations. Yin et al. referred to potential glial activation, neuronal death and impaired myelination^41^ as a result of reduced levels of plasma antioxidants such as bilirubin. Interestingly, Ehrenreich et al. has previously demonstrated that infusion with another antioxidant, human erythropoietin (EPO), the hematopoietic growth factor that induces the production of RBC, reduces cognitive deficits in patients with schizophrenia^42^.

Yin et al. emphasized that they were unable to control for potential confounders such as antipsychotics, body mass index (BMI), alcohol intake and smoking in their study and requested future analysis to be performed in antipsychotic naïve individuals with FEP^24^. In the present study the positive correlation between bilirubin and WMSIII-SS remained significant at 5 percent level in our sub group analysis of antipsychotic naïve individuals with FEP (see Supplementary Table S5 online). However, the correlation between bilirubin and WMSIII-LNS were only significant at 10 percent level (see Supplementary Table S5 online). We are hereby able to confirm the findings seen in chronic patients with schizophrenia in the study by Yin et al.^24^, but here in individuals with FEP, at the same time addressing the influence of antipsychotic medication, BMI, smoking and alcohol, as requested by the authors^24^.

Our finding of an inverse association between bilirubin and DUP is interesting since a longer DUP may be associated with more severe manifestations of schizophrenia^43^ and the importance of DUP as a descriptive variable in studies on individuals with FEP has previously been emphasized^44–46^. A longer DUP and lower bilirubin levels could potentially represent a longer exposure to stress in these individuals. However, a limitation regarding the entity of DUP is that the concept is heterogeneous regarding the frequency or severity of psychotic symptoms and quantified retrospectively^44^. The individuals with FEP in this study had a median DUP of 4.5 (2.0–18.0) months which is a relatively short duration^43^. Albeit reduced levels of bilirubin has been seen both in acute and chronic patients with schizophrenia, the clinical significance of a long DUP as well as potential dynamic changes in antioxidant status related to different phases of the disorder^9^ may compromise the generalizability of the cohort. However, the definition of DUP may vary and our DUP endpoint was initiation of any treatment targeting the psychotic symptoms. Several of the patients in the present study were recruited from highly specialised units for FEP treatment which may have enabled an earlier detection thus shortening the DUP although the results in GAF-F and GAF-S (Table 1) indicate a cohort with a high symptom load.

Symptom severity (PANSS) did not associate with bilirubin in our cohort, also in line with observations in the study by Yin et al.^24^. Some studies have reported positive associations between bilirubin levels and scores on PANSS Positive^36^, PANSS General^31^ and both PANSS Positive and General^47, 48^ scores. However, these associations were found in patients with schizophrenia with higher bilirubin levels compared with HC.

We found no significant differences in bilirubin levels between antipsychotic treated and antipsychotic-naïve individuals with FEP. This is in line with other studies indicating that the alterations in antioxidant functions seen in schizophrenia may be independent of antipsychotics^11, 12, 16, 20, 25^. However, some studies saw a decrease in bilirubin levels during antipsychotic treatment response^16, 33, 36, 48^ which was suggested to be mediated by its antioxidative effect^49^. Once again, this was seen in studies that reported higher levels of bilirubin in patients with schizophrenia compared with HC.

The finding of a correlation between bilirubin and age seen in HC, but not in FEP, is in concordance with the age-related changes in bilirubin seen in patients with chronic schizophrenia^20^. However, albeit not significant, the median age for HC was lower compared with individuals with FEP. Thus, there was no need to control for this parameter.

The lack of difference in bilirubin levels in patients on or off tobacco seen in this study is in line with some previous findings^9, 11, 12, 20, 26^. Even if cigarette smoking has been associated with oxidative stress and reducing antioxidants such as bilirubin^50–52^, it has also been suggested that the alterations in AODS seen in patients with schizophrenia—which are known to have a higher smoking prevalence than the general population—cannot be accredited to smoking alone^25^.

The additional comparisons of liver markers exclude the possible bias of antipsychotics and substance abuse, which both causes liver function alterations with altering bilirubin levels^53^. B-Hb and s-cobalamin are relevant to bilirubin levels as they are markers related to RBC and aberrations could also be indicative of an imbalanced diet. The latter may affect plasma antioxidants of dietary origin, thereby possibly influence levels of bilirubin due to synergistic protection and cooperative actions of different plasma antioxidants^20^. However, an imbalanced diet may not alone explain reduced levels of antioxidants seen in schizophrenia^54, 55^. P-albumin, another plasma antioxidant, is the major transporter protein of bilirubin and the concentration of albumin may influence bilirubin levels. Some of the studies that reported reduced bilirubin levels in schizophrenia also saw a concomitant decrease in albumin^9, 12, 25^. There were no indications of possible biases related to the abovementioned confounding factors in the present study.

Yin et al. requested future studies to include correlations of other antioxidants and cognition in schizophrenia^24^. To address this, we performed an additional explorative analysis of p-albumin and working memory. Interestingly, p-albumin correlated with WMSIII-SS both in the full FEP cohort (see Supplementary Table S8 online) and in the sub group analysis of the antipsychotic naïve individuals with FEP (see Supplementary Table S9 online). In line with the findings of bilirubin seen both in this study and in the abovementioned study by Yin et al., no such correlations were seen in HC (see Supplementary Table S10 online). Altogether, this further strengthens the notion that the antioxidant deficits seen in schizophrenia may be implicated in the memory impairments.

Given the complex nature of the disorder, the discrepancies of both lower and higher levels of bilirubin seen in the literature could indicate a nonlinear and multifactorial relation between the pathophysiology of schizophrenia and bilirubin and the disorder may both be the cause and the effect of the fluctuations in bilirubin^56, 57^. There seems to be an inflammatory brain mechanism associated with both the studies reporting higher and lower bilirubin levels in schizophrenia; high bilirubin levels may directly, and low levels may indirectly (by the depletion in AODS) result in ROS production, neuro-inflammation and cell apoptosis^24, 56^.

The discrepancies seen in the literature could also be due to heterogeneity between studies, e.g. regarding different fractions of bilirubin. Ethnicity could potentially introduce a bias^58^ and many of the studies on bilirubin have been conducted on different ethnic populations. To our knowledge this is the first study of a Swedish cohort. The heterogeneity of schizophrenia^59, 60^, different disorders on the schizophrenic spectra^61^ and dynamic changes related to different phases of the disorder^9^ may also serve as possible explanations to the discrepancies.

The major strength of this study is its design of a well characterized cohort of individuals with FEP and demographically matched HC. In the individuals with FEP, a majority were naïve to antipsychotics, the rest only short-time medicated, and about a third were unmedicated in regard to all psychotropic drugs. To our knowledge this is the first study on individuals with FEP presenting correlations of bilirubin with memory and DUP. We were able to control for a variety of confounding demographic factors, smoking and antipsychotic mediation as well as psychotropic drugs. We also excluded individuals with alcohol and substance abuse. We believe that this well characterized cohort of individuals with FEP has a unique potential to add new knowledge to this field. Even if we had no data on the presence of metabolic syndrome, another potential confounding factor relevant to bilirubin levels in patients with schizophrenia^62^, BMI did not differ between individuals with FEP and HC and the median value of BMI and were within normal limits in both cohorts. Diurnal variation and physical activity may affect bilirubin levels and efforts were made to standardise the study procedure^39^.

A limitation of the present study is the relatively small sample size and the fact that the HC were recruited by advertisement. FEP is an intermediate diagnosis and even if the majority of patients were diagnosed with schizophrenia spectrum disorders in 1.5-year re-evaluation, caution is always needed when extrapolating FEP to schizophrenia due to the heterogeneity of these patients. Although there were no significant gender differences between groups, two-thirds of the individuals with FEP in this study were males. It is however not likely that the finding of reduced bilirubin levels in individuals with FEP is due to any selection bias. On the contrary, previous studies have shown less reduced levels of bilirubin in sub group analysis of male individuals with FEP compared with male HC versus female individuals with FEP compared with female HC^12, 25^.

Bilirubin has been suggested to be a potential biomarker candidate^56, 63^ with possible therapeutic implications^12, 13, 64^. Future studies could quantify the different fractions of bilirubin for comparison with its oxidative products, biopyrrins, since a buffer system of sorts between the heme metabolite and ROS has been suggested^56^. Our findings support the notion of increased oxidative stress in early-stage schizophrenia, in turn confirming the preliminary findings^24^ of correlations with the memory impairments that accompanies the disorder.

## Supplementary Information

Supplementary Information.
