# Supplementary material for: Plasma bilirubin levels are reduced in first-episode psychosis patients and associates to working memory and duration of untreated psychosis
Source: Sci Rep. 2021 Apr 6;11:7527. doi: 10.1038/s41598-021-87096-z (PMC8024299; doi:10.1038/s41598-021-87096-z)
Supplement: Supplementary file 1 — Supplementary Information. [file 41598_2021_87096_MOESM1_ESM.pdf]

Plasma bilirubin levels are reduced in first-episode psychosis patients and associates to working memory and duration of untreated psychosis

Meneca Becklén [a], Funda Orhan [a], Fredrik Piehl [b], Simon Cervenka [c], Carl M. Sellgren [a, c], Lena Flyckt [c], Sophie Erhardt [a*], Helena Fatouros-Bergman [c]

[a] *Department of Physiology and Pharmacology, Karolinska Institutet, Stockholm, Sweden*
[b] *Neuroimmunology Unit, Department of Clinical Neuroscience, Karolinska Institutet, Stockholm, Sweden*
[c] *Centre for Psychiatry Research, Department of Clinical Neuroscience, Karolinska Institutet, & Stockholm Health Care Services, Region Stockholm, Stockholm, Sweden*

*Corresponding author at: Department of Physiology and Pharmacology, Karolinska Institutet, SE-171 77 Stockholm, Sweden
E-mail address: sophie.erhardt@ki.se (S. Erhardt)

Characteristic	Median [Interquartile range] (n)<br>**Healthy controls (n=20)**	**FEP patients (n=39)**	**p-value**
Gender (M:F)	10:10 (20)	24:15 (39)	0.396[a]
Age (years)	24.5 [21.5-27.5] (20)	27.0 [22.0-33.0] (39)	0.121[b]
BMI (kg m-2)	21.9 [20.8-23.9] (19)	21.9 [20.6-25.2] (37)	0.810[b]
Weight (kg)	69.3 [61.5-81.9] (16)	70.0 [62.0-76.8] (37)	0.870[b]
Length (cm)	173 [169-185] (17)	176 [169-180] (37)	0.970[b]
Tobacco (Y:N)	0:20; 0% (20)	12:24; 33% (36)	0.004[a]*
DUP (months)	-	4.0 [1.0-12.0] (35)	-
Medication (Y:N)			-
Antipsychotics	-	16:23; 41% (39)	-
Benzodiazepines	-	11:28; 28% (39)	-
Zopiclone	-	10:29; 26% (39)	-
Antidepressants	-	4:35; 10% (39)	-
Phenothiazine derivatives	-	11:28; 28% (39)	-
Antiepileptic’s	-	1:38; 2,6% (39)	-
Combination of > 2 drugs	-	15:24; 38% (39)	-
Totally drug-naive	-	12:27; 31% (39)	-
PANSS			
Positive	-	19 [14-23] (39)	-
Negative	-	13 [10-19] (39)	-
General	-	37 [28-45] (39)	-
Total	-	72 [60-83] (39)	-
Anxiety	-	4 [3-5] (39)	-
Depression	-	3 [2-4] (39)	-
Level of functioning			
GAF S	-	31 [28-39] (39)	-
GAF F	-	40 [35-50] (39)	-
GAF S HVLY	-	67 [50.5-75] (36)	-
GAF F HVLY	-	67.5 [57.5-79.5] (36)	-
CGI Score	-	4.0 [3.0-5.0] (39)	-

Supplementary Table S1. Demographic and clinical characteristics of all individuals with detectible bilirubin values after exclusion of an outlier (a HC) with a bilirubin value of 52 µmol/l.

Abbreviations***:*** FEP, first-episode psychosis; M:F, Male:Female; Y:N, Yes:No; (n), number; BMI, body mass index; DUP, Duration of untreated psychosis; PANSS, Positive and Negative Syndrome Scale; GAF S, Global Assessment of Functioning Symptoms; GAF F, Global Assessment of Functioning Functions; HVLY, highest value last year; CGI, Clinical Global Impression. [a]Pearson’s chi-squared test [b]Mann-Whitney U-test, two-sided. *p-value <0.05

Cognitive domain	Test	Mean/Median [±S.E.M.]/[IQR] Healthy controls (n=21)	FEP patients (n=41)	p-value
Verbal learning	HVLT-R	29 [±0.59]	24 [±0.78]	<0.0001[A]*
Working memory	WMSIII-SS	19 [±0.57]	16 [±0.50]	0.0024[B]*
Working memory	WMSIII-LNS	16 [±0.54]	13 [±0.50]	0.0057[B]*
Speed of processing	TMT-A	23 [18-26]	28 [22-37]	0.0113[C]
Speed of processing	BACS-SC	62 [±1.6]	47 [±2.0]	<0.0001[A]*
Speed of processing	Fluency-AN	26 [±1.2]	22 [±0.9]	0.0093[B]

Supplementary Table S2. Comparison of test results in the cognitive domains verbal learning, working memory and speed of processing between HC and individuals with FEP.
Abbreviations: FEP, first-episode psychosis; IQR, interquartile range; S.E.M., standard error of the mean; (n), number; HVLT-R, Hopkins Verbal Learning Test - Revised; WMSIII-SS, Wechsler Memory Scale-3rd Edition - Spatial Span; WMSIII-LNS, Wechsler Memory Scale-3rd Edition – Letter-Number Span; TMT-A, Trail Making Test – Part A; BACS-SC, Brief Assessment of Cognition in Schizophrenia - Symbol Coding; Fluency-AN, Category Fluency - Animal Naming. [A]Satterthwaite t-test (parametric), unequal variances. [B]Student's unpaired t-test (parametric), pooled variance. [C]Mann-Whitney U-test (non-parametric), two-sided. *Bonferroni-corrected, α-value= 0.00833

Characteristic	r-value	(n)	p-value
Gender (M:F)	0.25	39	0.129
Age (years)	0.09	39	0.571
BMI (kg m-2)	0.05	37	0.764
Weight (kg)	0.12	37	0.467
Length (cm)	0.21	37	0.215
Tobacco (Y:N)	-0.06	36	0.728

Supplementary Table S3. Spearman's rank correlation of plasma bilirubin levels with demographic factors and tobacco use in individuals with FEP.
Abbreviations: FEP, first-episode psychosis; (n), number; M:F, Male:Female; Y:N, Yes:No; BMI, body mass index.

Characteristic	r-value	(n)	p-value
Gender (M:F)	0.44	20	0.050
Age (years)	0.60	20	0.005*
BMI (kg m-2)	0.12	19	0.626
Weight (kg)	0.27	16	0.313
Length (cm)	0.38	17	0.128

Supplementary Table S4. Spearman's rank correlation of plasma bilirubin levels and demographic factors in healthy controls.
Abbreviations: (n), number; M:F, Male:Female; Y:N, Yes:No; BMI, body mass index *p-value <0.05

Cognitive test	r-value	(n)	p-value
Working Memory (WMSIII-SS)	0.45	22	0.034*
Working Memory (WMSIII-LNS)	0.36	22	0.098

Supplementary Table S5. Spearman's rank correlation of plasma bilirubin levels and working memory in antipsychotic naïve individuals with FEP. Abbreviations: FEP, first-episode psychosis; (n), number; WMSIII-SS, Wechsler Memory Scale-3rd Edition - Spatial Span; WMSIII-LNS, Wechsler Memory Scale-3rd Edition – Letter-Number Span. *p-value <0.05

Cognitive test	r-value	(n)	p-value
Working Memory (WMSIII-SS)	-0.21	20	0.383
Working Memory (WMSIII-LNS)	0.15	20	0.518

Supplementary Table S6. Spearman's rank correlation of plasma bilirubin levels and working memory in healthy controls.
Abbreviations: (n), number; HVLT-R; Hopkins Verbal Learning Test - Revised; WMSIII-SS, Wechsler Memory Scale-3rd Edition - Spatial Span; WMSIII-LNS, Wechsler Memory Scale-3rd Edition – Letter-Number Span; BACS-SC, Brief Assessment of Cognition in Schizophrenia - Symbol Coding.

	Mean/Median [±S.E.M.]/[IQR]		
**Blood markers**	**Healthy controls**	**FEP patients**	**p-value**
	(n=21)	(n=42)	
p-albumin (g/L)	45 [41-47] (19)	46 [44-48] (39)	0.55[A]
s-cobalamin (pmol/L)	210 [190-290] (20)	275 [200-330] (38)	0.09[A]
b-Hb (g/l)	133 [±3.29] (20)	139 [±2.41] (39)	0.15[B]
p-ALT (µkat/L)	0.28 [0.22-0.38] (20)	0.37 [0.22-0.82] (39)	0.09[A]
p-AST (µkat/L)	0.29 [0.24-0.36] (20)	0.35 [0.26-0.56] (39)	0.05[A]
p-GGT (µkat/L)	0.1 [0.1-0.2] (20)	0.1 [0.1-0.2] (39)	0.79[A]

Supplementary Table S7. Comparisons of other blood markers between healthy controls and individuals with FEP.
Abbreviations: IQR, interquartile range; S.E.M., standard error of the mean; FEP, first-episode psychosis; (n), number; p, plasma; s, serum; b, blood; Hb, haemoglobin; ALT, alanine transaminase; AST, aspartate transaminase; GGT, gamma-glutamyl transferase. [A]Mann-Whitney U-test (non-parametric), two-sided. [B]Student's unpaired t-test (parametric), pooled variance.

Cognitive test	r-value	(n)	p-value
Working Memory (WMSIII-SS)	0.36	38	0.026*
Working Memory (WMSIII-LNS)	0.10	38	0.533

Supplementary Table S8. Spearman's rank correlation of plasma albumin levels and working memory in all individuals with FEP.
Abbreviations: FEP, first-episode psychosis; (n), number; WMSIII-SS, Wechsler Memory Scale-3rd Edition - Spatial Span; WMSIII-LNS, Wechsler Memory Scale-3rd Edition – Letter-Number Span. *p-value <0.05

Cognitive test	r-value	(n)	p-value
Working Memory (WMSIII-SS)	0.50	22	0.019*
Working Memory (WMSIII-LNS)	0.30	22	0.179

Supplementary Table S9. Spearman's rank correlation of plasma albumin levels and working memory in antipsychotic naive individuals with FEP.
Abbreviations: FEP, first-episode psychosis; (n), number; WMSIII-SS, Wechsler Memory Scale-3rd Edition - Spatial Span; WMSIII-LNS, Wechsler Memory Scale-3rd Edition – Letter-Number Span. *p-value <0.05

Cognitive test	r-value	(n)	p-value
Working Memory (WMSIII-SS)	-0.1626	19	0.5059
Working Memory (WMSIII-LNS)	0.1492	19	0.5422

Supplementary Table S10. Spearman's rank correlation of plasma albumin levels and working memory in healthy controls.
Abbreviations: (n), number; HVLT-R; Hopkins Verbal Learning Test - Revised; WMSIII-SS, Wechsler Memory Scale-3rd Edition - Spatial Span; WMSIII-LNS, Wechsler Memory Scale-3rd Edition – Letter-Number Span; BACS-SC, Brief Assessment of Cognition in Schizophrenia - Symbol Coding.
